# Histopathological study of the mite biting* (Dermanyssus gallinae)* in poultry skin

**Published:** 2012

**Authors:** Rahim Hobbenaghi, Mousa Tavassoli, Manochehr Alimehr, Sara Shokrpoor, Mohammad Ghorbanzadeghan

**Affiliations:** 1 *Department of Pathobiology, Faculty of Veterinary Medicine, Urmia University, Urmia, Iran; *; 2 * Department of Clinical Sciences, Faculty of Veterinary Medicine, Urmia University, Urmia, Iran; *; 3 * Department of Pathobiology, Faculty of Veterinary Medicine, Tehran University, Tehran, Iran.*

**Keywords:** *Dermanyssus gallinae*, Poultry, Pathologic changes, Mite, Skin

## Abstract

The red mite of poultry, *Dremanyssus gallinae*, is the most important hematophagous ectoparasite of poultry. In this study, pathologic changes of its biting on the poultry skin have been investigated. Thirty-two (Control = 16 and Treatment = 16) four weeks old Ross broilers (308) were infested with the mite on skin of hock joins. Samples were collected after 1, 24, 72 hours and 10 days. The skin samples were fixed in 10% buffered formalin and histological sections were prepared using routine Hematoxylin & Eosin staining method. Results showed that in all cases, except within first hour of infestation, lymphocytic infiltration was always a constant pathologic feature. Necrosis of feather's follicles was a prominent pathologic feature ensued due to vascular disturbances and resulted in loss of feather. Hyperkeratosis, parakeratosis and acanthosis were observed after 72 hours. These findings reveal that mite biting induces local epidermal hyperplasia.

## Introduction

Mites have long been recognized as a cause of dermatitis. Mites damage the skin with their feeding habits and are vectors for a number of important diseases. These parasites are easily distinguished from other arachnids by the possession of a distinct gnathosoma (capitulum with the mouthparts) and the lack of a division between the abdomen and cephalothorax.^1^

The poultry red mite, also named chicken mite, *Dermanyssus gallinae*, (Acari: Dermanyssidae), is the most important hematophagous ectoparasite of domestic poultry. Infestations are prevalent worldwide and they are common among poultry houses in both developed and developing countries.^2^ Breeding cycle of* D. gallinae* is very short (7- 10 days). Infestation with this parasite induce anemia, irritation, decrease in egg production and feather pecking.^3^^, ^^4^

*Dermanyssus gallinae* is also known to be a potential vector for various poultry and other livestock pathogens, including *Salmonella* *spp*., chicken pox virus, avian spirochetes, Newcastle disease virus and agents of erysipelas, fowl typhoid, cholera and eastern equine encephalitis virus.^5^^-^^12^

Feeding of this mite cause damage especially to chest and legs of affected birds manifested by skin inflammation^13^and itching.^14^ The newly hatched chickens die of severe infestation.^15^

Occasionally, *D. gallinae* causes dermatitis, severe pruritus and a nuisance to people working at heavily infested poultry houses.^2^^,^^16^^,^^17^ Hewitt *et al* in 1971 realized that response to the mite is severe and skin redness, edema and infection with pyogenous bacteria will ensue.^18^ In addition to birds, mammals ,including human being, could suffer from it.^18^ In a human case study, dermatitis, skin edema, itching, erosion and redness have been reported.^19^ In a dermatologic clinic, more than 5% of itching cases were infected with *D. gallinae* that lead to edema of skin and Eustachian tube.^19^^,^^20^

The aim of the present study was to evaluate pathologic changes of poultry skin caused by *D. gallinae* biting in various periods of times.

## Materials and Methods

**Sample Collection and identification. **The mites were collected from Urmia poultry farms and transferred to the Parasitology laboratory of Faculty of Veterinary Medicine, Urmia University (Iran). They were identified to species based on Wall and Shearer.^21^The mites were kept in room temperature with 80% relative humidity inside desiccators until use.

**Infestation of chicks with the red mite. **Thirty-two 4week olds healthy broilers (Ross, 308) were prepared from Urmia poultry farms. They were kept in metallic cages and allowed to acclimatize for 7 days. The food and water were provided *ad libitum*. The macroscopic evaluations confirmed that the chicks were ectoparasite free. They were divided into the control (n =16) and the treatment (n =16) groups. Then, each group was further randomly divided into four subgroups (4 chicks in each) based on infestation time interval. To infestation of the chicks the mites were contacted with skin by sewn fabric tubule. 

At 1, 24, 72 hours and 10 days after infestation, chicken were euthanized and the infested skin were sampled.

**Histopathologic studies. **The specimens were collected, fixed in 10% buffered formalin and processed for paraffin embedding, sectioned in 5 µm thin slices and stained with Hematoxylin & Eosin (H&E) and examined by pathologist.

## Results

The histopathologic results were blindly examined by pathologist. The samples taken at 1 hour after infestation showed subcutaneous edema and congestion. 

At 24 hours, subcutaneous edema, severe congestion of hypoderma, scattered lymphocyte infiltration foci and necrosis of feather follicles. 

At 72 hours, subcutaneous edema, severe congestion, lymphocyte infiltration foci, necrosis of feather follicles, heterophilic infiltration, hyperkeratosis, parakeratosis and acanthosis and at 10 days, subcutaneous edema, severe congestion, extensive lymphocyte infiltration foci, hydropic degeneration of feather follicles with presence of vacuoles inside the cells, necrosis of feather follicles, heavy feather loss, hyperkratosis, parakeratosis, acanthosis and thrombosis were observed. 

**Fig. 1:**
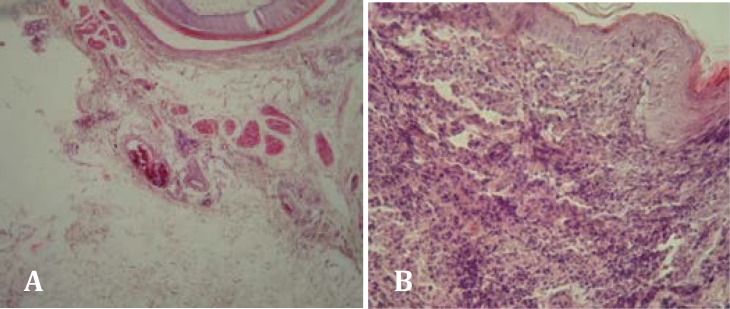
**A.** Edema and congestion in subcutaneous (H&E, 40×). **B.** Acanthosis and lymphocytic infiltration increasing of epidermal layers (H&E, 200×).

**Fig.2:**
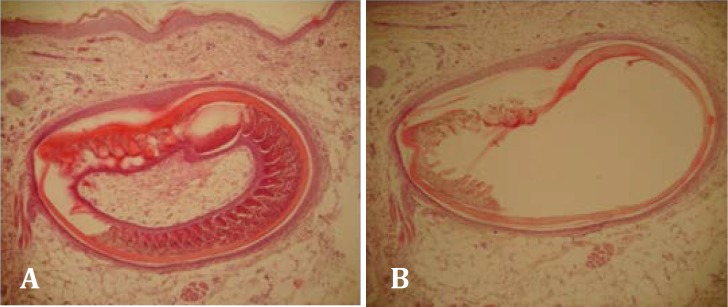
**A.** Hydropic degeneration in a feather follicle (H&E, 40×). **B. **Hydropic degeneration and necrosis in a feather follicle (H&E,40×).

**Fig.3:**
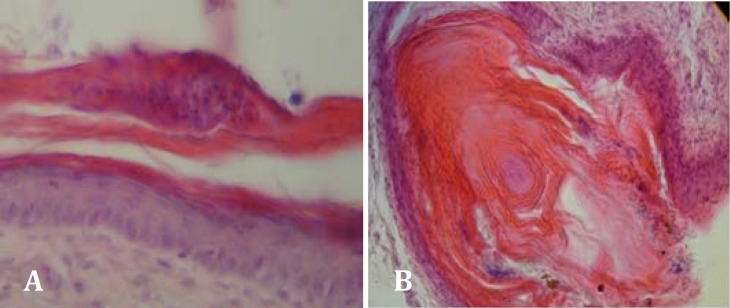
**A.** Parakeratosis, remaining of nucleus in cornium Layer (H&E, 400×). **B.** Hyperkeratosis, increasing of cornified layer (H&E, 100×).

**Fig.4:**
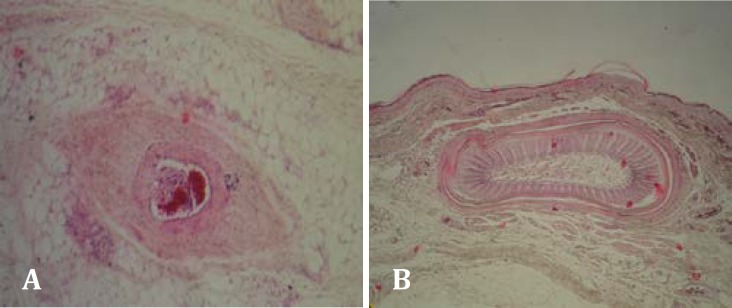
**A.** Vascular thrombosis in subcutis in 10 days group (H&E, 100×). **B.** Normal skin layers, a feather follicle in control group (H&E, 40×).

Overall, in comparison with control group (Fig. 4B) all infected cases had subcutaneous edema and congestion (Fig. 1A), 12 cases (75%) had lymphocyte infiltration foci and necrosis of feather follicles (Figs. 1B, 2A, and 2B), 8 cases (50%) had hyperkeratosis, parakeratosis and acanthosis. (Figs. 1B, 3A, and 3B), 4 cases (25%) had feather follicles loss and thrombosis (Fig. 4A). 

Histiocytes were seen at least in 50% of lymphocytic foci under infested epidermis.

## Discussion

All mites may produce pruritus or allergic reactions through salivary proteins deposited during feeding.^22^ Generally, lesions caused by mites are pruritic, somewhat erythematous eruptions composed of papules that may or may not be associated with a wheal.^1^

Vertebrate immune responses to mites are best understood in mammal-tick systems, where immunity is acquired through a combination of humoral and cell mediated responses.^23^ Cell mediated responses cause inflammation at the site of the bite, which prevents the mite from obtaining a blood meal. Humoral responses involve antibodies that bind with proteins in the mite’s saliva and prevent it from feeding effectively, or that inhibit nutrient absorption in the mite’s gut. However, mites are not passive targets for host immune defenses.^24^

Immunological studies of bird-mite systems are less common, but they have parallels with mammal-tick immune systems. Devaney and Augustine found that domestic hens developed a humoral antibody after several days of being infested with tropical fowl mites, and appearance of the antibody was correlated with a reduction in population growth rate of the mites.^25^ Gwinner *et al.* used afield test to show that starlings in nests with high loads of red fowl mites had a greater immune response when injected with a foreign protein than did those from nests with low mite loads, thus suggesting that the nestlings’ immune systems were ‘primed’ to withstand the effects of mite feeding.^26^

In this study lymphocytic infiltration in most (75%) samples indicates that the released materials by mites into the subcutaneous induce immune response and the longer the time, the more the lymphocytic infiltration. This result supports the works of Sokol and Rotkiewicz who reported 100% lymphocytic infiltration in all carcasses of a layers farm.^27^ The difference in rate between these two studies can be attributed to of the longer contact time of the mites with host skin in latter study. Extensive infiltration and migration of lymphocytes also has been reported in skin of cases infected with ornithonyssussylviarum.^28^ Mites can induce injury in subcutis vessels while biting and cause vascular thrombosis and therefore disturbances of subcutaneous blood supply. These circulatory disturbances can explain the pathologic changes in leather follicles such as hydropic degeneration and necrosis. It should be noted that extensive vascular network in subcutis prevents large and visible necrosis in dermis, whereas it could cause limited degeneration and necrosis in a sensitive growing organs such as follicles.

In this study, presence of subcutaneous edema in about all cases indicates an increasing vascular permeability due to vascular damage from blood sucking activity of the mite. As Hewitt *et al*. and Sokol and Rotkiewicz showed salivary materials of mites induced inflammatory responses and subcutaneous involvement, therefore this edema is an introduction to inflammatory process.^18^^,^^27^

Local hyperkeratosis, the hyperplasia of germinal layer of epidermis, acanthosis and also parakeratosis were observed in most samples after 72 hours of infestation. Presence of hyperplasia in germinal layer of epidermis and their subsequent events (acanthosis, hyperkeratosis and parakeratosis) shows that mite biting induces proliferation of epidermal cells and local hyperplasia. These findings support works of Sokol and Rotkiewicz and Shanta *et al*. which reported hyperplasia, hypertrophy, hyperkeratosis and acanthosis in *D. gallinae* and *knemidocoptes mutans*, respectively.^27^^-^^30^

Mignon and Losson reported skin lesions and hair loss in frontal, cervical and caudal regions of horses being in contact with poultries infected with *D.gallinae*.^31^ Regarding this study and aforementioned studies it could be concluded that mites could cause a broad spectrum of dermal and hypodermal lesions in various species regardless of their genders.
